# Customized composite veneers from a totally digital workflow: A case report

**DOI:** 10.1002/ccr3.3104

**Published:** 2020-07-15

**Authors:** Luca Ortensi, Tommaso Vitali, Marco Ortensi, Luca Lavorgna, Maria Laura Strocchi

**Affiliations:** ^1^ DDS Department of Prosthodontics University of Catania Catania Italy; ^2^ DDS Private Practice Perugia Italy; ^3^ CDT Private Practice Bologna Italy; ^4^ DDS Private Practice Telese Terme Italy; ^5^ DDS Private Practice Bologna Italy

**Keywords:** composite laminate veneers, dental aesthetics, digital smile system, rubber dam, treatment planning

## Abstract

A treatment plan based on the use of a preview software can offer the possibility to rapidly communicate with the patient. Fully digital workflow allows for making several objects at the same time in a precise and cost‐efficient manner.

## Graphical abstract

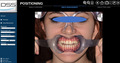

## 1 INTRODUCTION

The evolution of composite materials and enamel‐dentin adhesive techniques has profoundly changed the restorative approach of dental elements affected by caries or imperfections both in the front and back of the oral cavity.^1^

For the front teeth, direct aesthetic restoration, requiring minimal preparation of the tooth, is a widely used therapy, is easily applicable, and is affordable. However, these types of restorations are more subject to wear, microinfiltration, and fractures, and, in some clinical situations, the indirect technique with composite veneers is an excellent treatment option to counter those phenomena thanks to their greater resistance.^2^, ^3^, ^4^ Composite veneers can also be considered a valid therapeutic alternative for treatment of front teeth aesthetic abnormalities, as well as those in ceramic material^5^, ^6^ due to the improved properties of the materials. New composites consist of about 66% inorganic fillers by volume. This seems to improve their mechanical properties, with a bending resistance of 120‐160 MPa and a modulus of elasticity of 8.5‐12 GPa.^7^ Composite veneers offer some advantages compared to ceramic restoration: more affordable, both for the clinic and the patient, less intrinsically fragile, and easier to repair.^8^

The indications for their use can be summarized into 3 different groups^9^:

- Dental discoloring resistant to whitening procedures;
- Need for significant morphological changes to front teeth;
- Extensive restoration of compromised front teeth.

The patient's aesthetic preferences might also be an important element for choosing the right treatment plan, such as the size and shape of front dental elements. Today's professional tends to choose the best treatment in accord with the patient, achieving a so‐called “therapeutic compromise.” Use of 2D software for processing images, for creating virtual planning, represents a valid support for the dialogue with the patient to achieve the preset goal.^10^ Among the various digital smile processing systems, one of the most common is the Digital Smile System.^11^

This software can digitally plan the aesthetic and functional rehabilitation of the smile,^12^ starting from several pictures taken of the face with a codified procedure associated with glasses for calibrating the software.^13^

The possibility of making veneers that are in line for size and shape with what the patient saw in the virtual smile simulation must be an achievable goal today.

The purpose of this article is to show, through the description of a clinical case, a fully digital workflow in which the digital preview of the smile goes as far as fabrication of an aesthetic composite part corresponding to the patient's aesthetic demands.

### 1.1 Description of the clinical case

**FIGURE 1:**
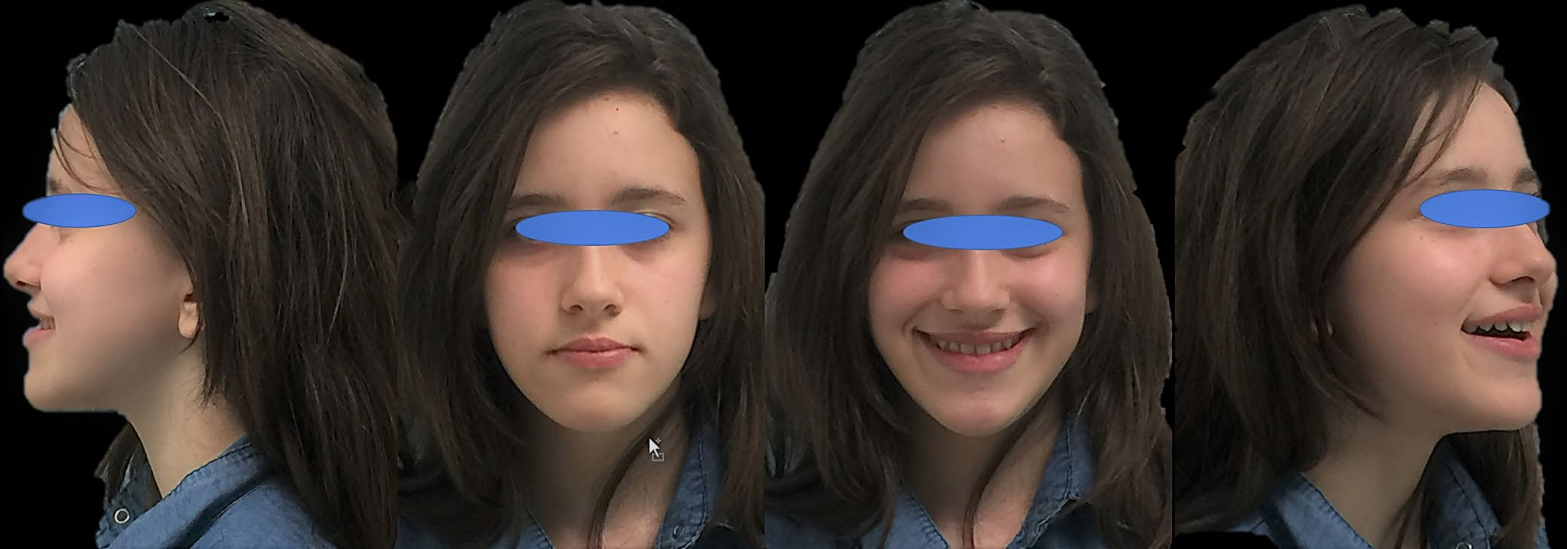
Appearance of the patient's face at the start of therapy. Notice the abnormal skeletal class

**FIGURE 2:**
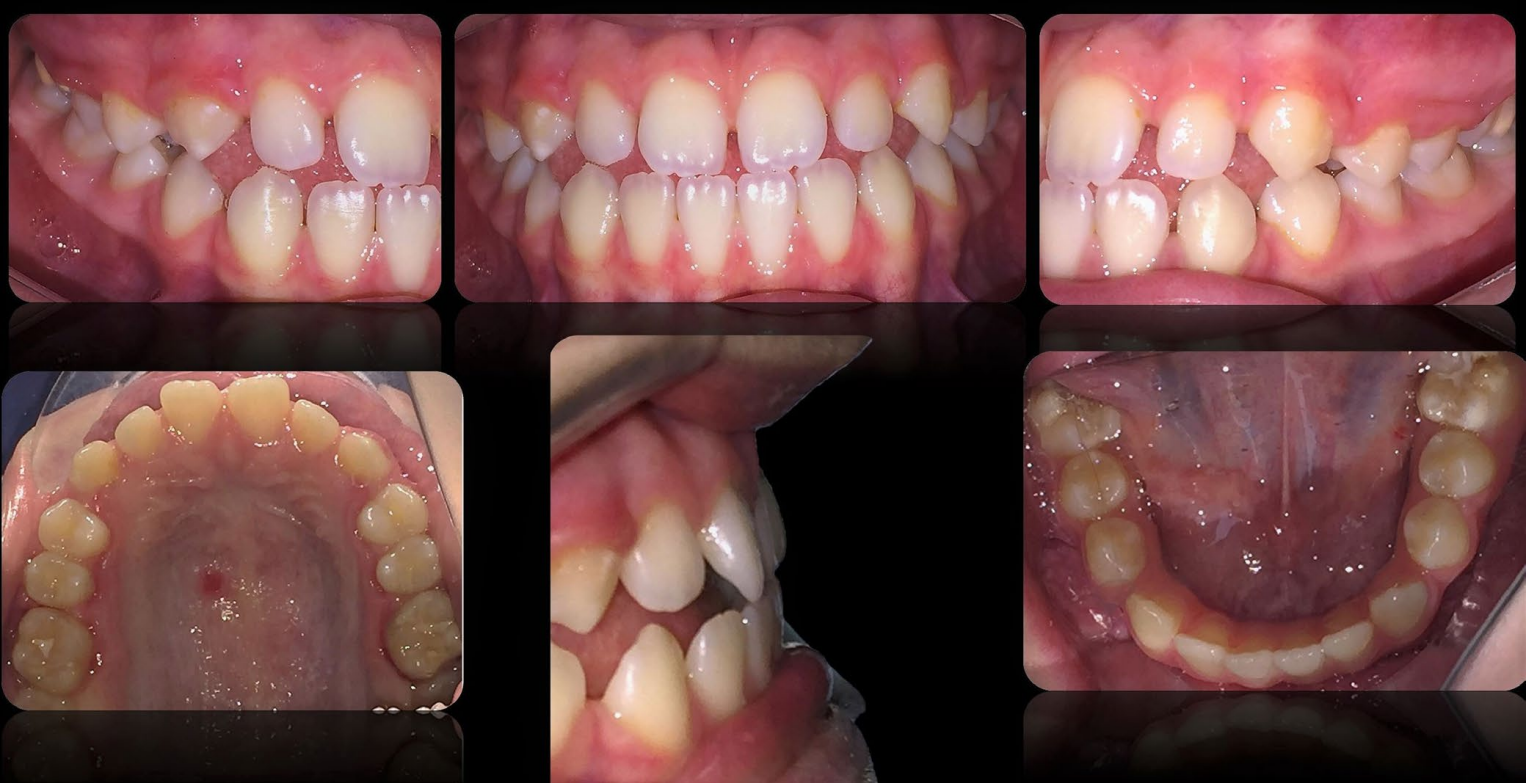
Intraoral appearance of the arches with detail of the overjet

An 18‐year‐old patient AB presents to our observation requesting aesthetic improvements of her smile and in particular closure of the gaps in the upper front group. The clinical examination of the patient showed a class III dental and skeletal class with a reduced overjet, good periodontal and temporomandibular joint health (Figures 1 and 2).

X‐rays and latero‐lateral X‐rays were performed to assess the dental status for the cephalometric examination necessary to confirm the alteration of the ratio between the bone foundations.

### 1.2 The following therapy was proposed to the patient

- Surgical orthodontic‐orthognathic treatment to restore the right ratio between the bone foundations to achieve a skeletal class I. With the preparatory orthodontic treatment for the orthognathic surgery, the upper frontal group is aligned, because it does not match with the dental arrangement.
- Indirect composite reconstructions were used to achieve ideal aesthetics of the smile through closure of gaps, without preparing the teeth.

The patient rejected surgery and instead opted for therapy not including orthognathic surgery, but would instead improve the aesthetic of her smile.

We proposed the patient therapy with aligners to improve the dental arrangement of her upper teeth, reiterating that it would be impossible to achieve a correction of the skeletal class. After dental alignment, indirect composite reconstructions would be applied without preparing the teeth.

After the patient accepted the proposed therapy, a preliminary procedure was implemented that included:

- Taking 2 digital pictures of the face according to a codified protocol and wearing dedicated glasses that have the double function of being a measurement instrument and an auxiliary to achieving a neutral head position. Both photographs were captured using a tripod, with the patient sits up straight and positions her head so that the Frankfurt plane is parallel to the horizon. The Nikon D300 camera, AF‐S VR Micro‐Nikkor 105mm f/2.8G IF‐ED lens, Nikon SB‐R 200 flash with LumiQuest pocket bouncer, on a Medical Close‐up Scorpion bracket, (Nikon Corporation, Japan) is positioned at the same height as the patient's face so that she can look straight into the lens, and the bipupillary plane is as parallel as possible to the horizontal plane. This method increases the reliability of multiple images acquisition as well as the accuracy in the subsequent virtual smile design process.

The first picture (F1) of the face was performed with spreaders, with semi‐discluded arches to correctly assess both the parallelism between the bipupillar and occlusal planes and the congruence of the median and interincisive lines.

**FIGURE 3:**
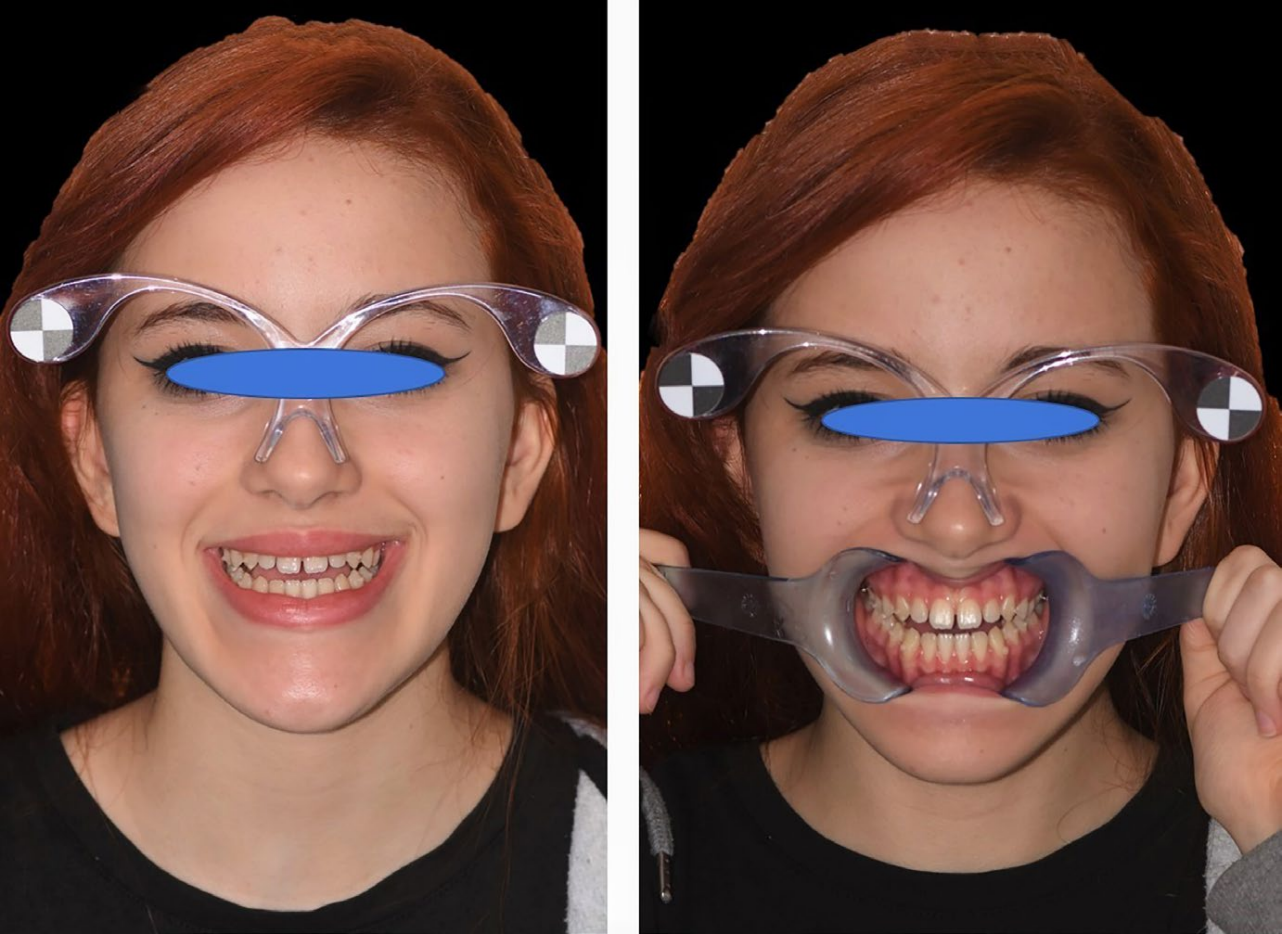
Photograph of the face done with dedicated glasses for calibration of the Digital Smile System software

The second photograph (F2) of the face was taken after removal of the spreaders and with the patient smiling, to assess the development of the incisal plane compared to the lower lip, as well as the width of the side corridors^14^, ^15^ (Figure 3).

**FIGURE 4:**
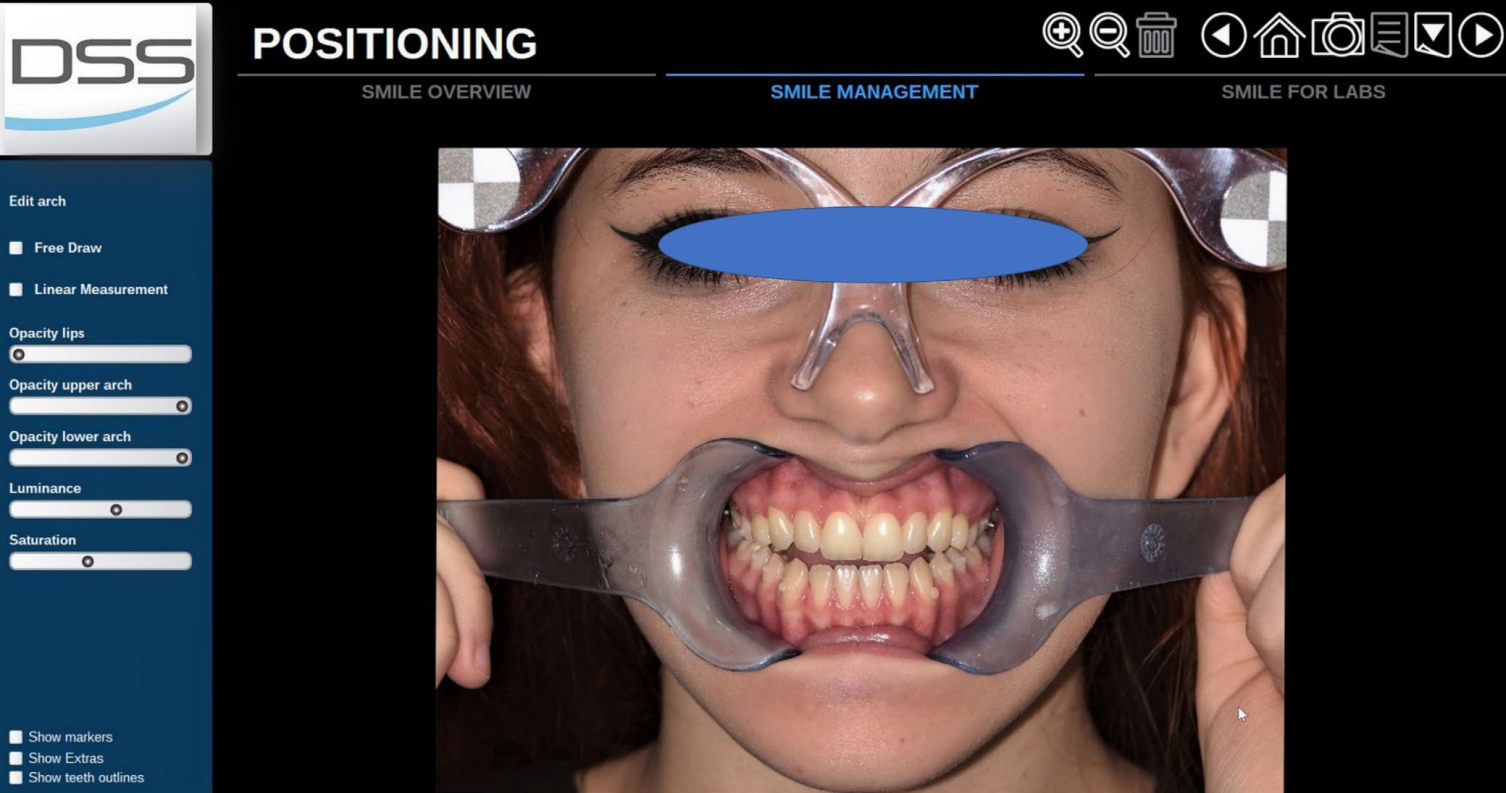
Preview of the patient's final aesthetic

**FIGURE 5:**
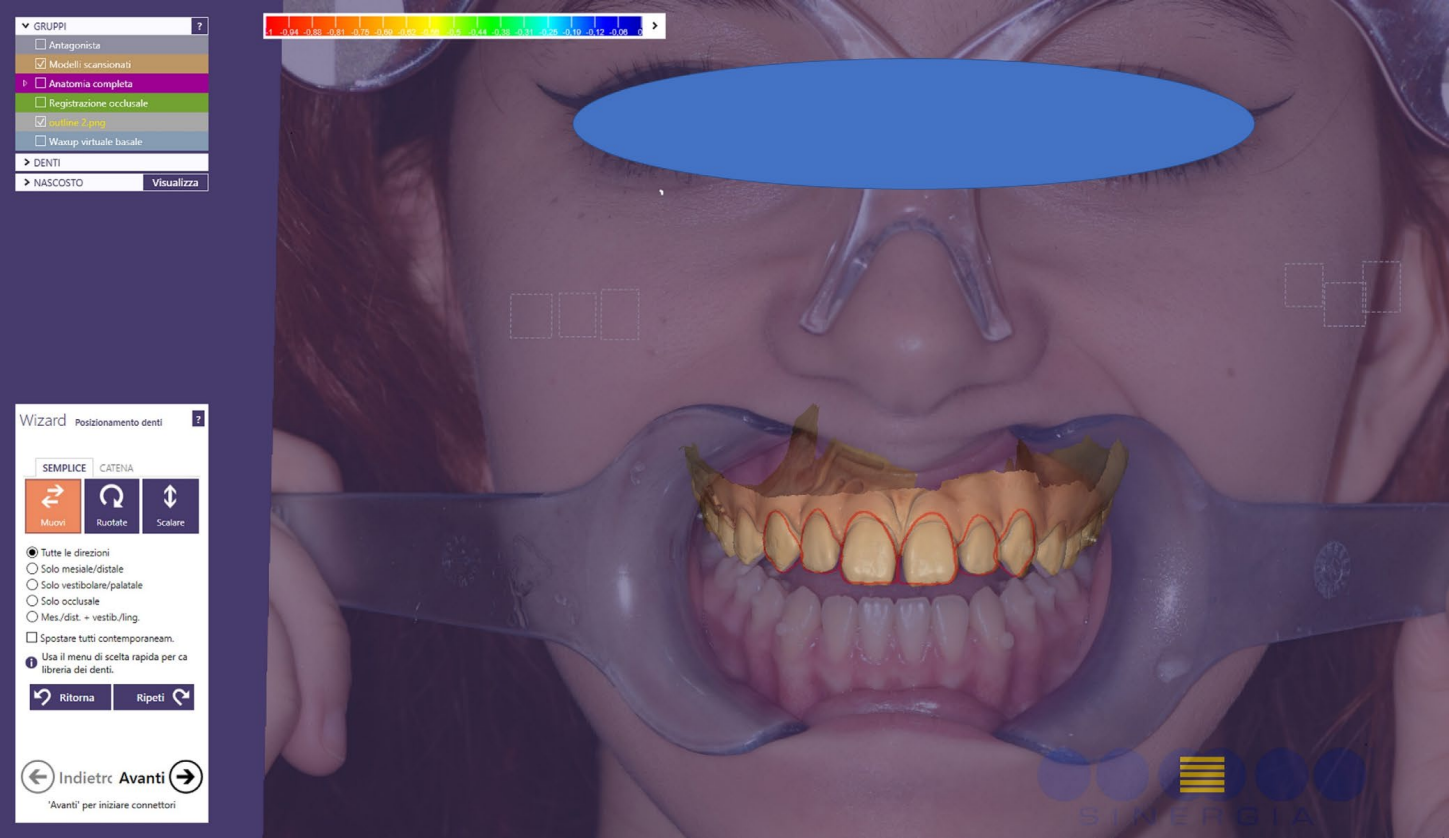
Overlap of the 2D image of the patient's smile with the 3D impression inside a 3D software

**FIGURE 6:**
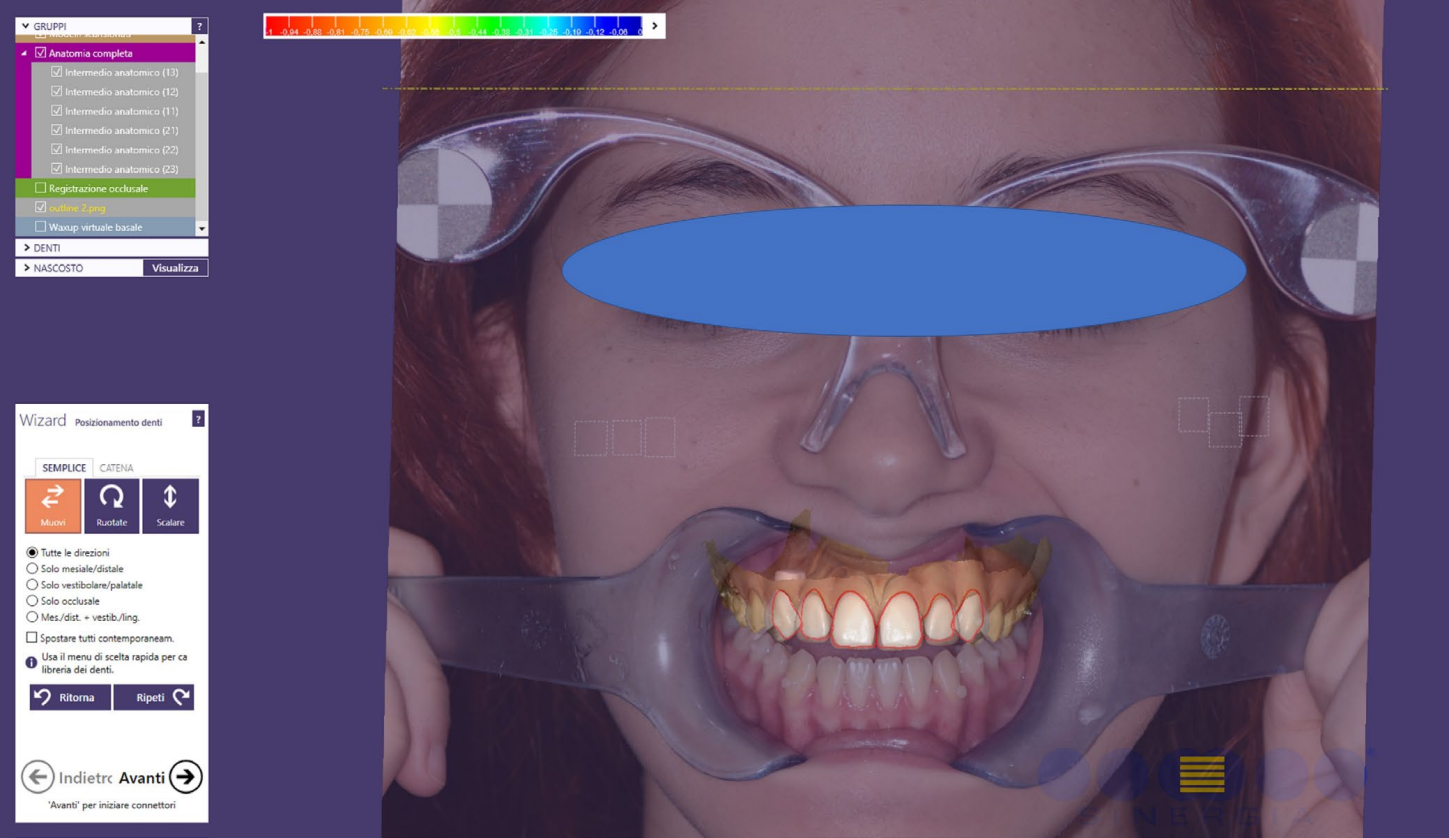
Virtual wax‐up made following the outlines of the preview

**FIGURE 7:**
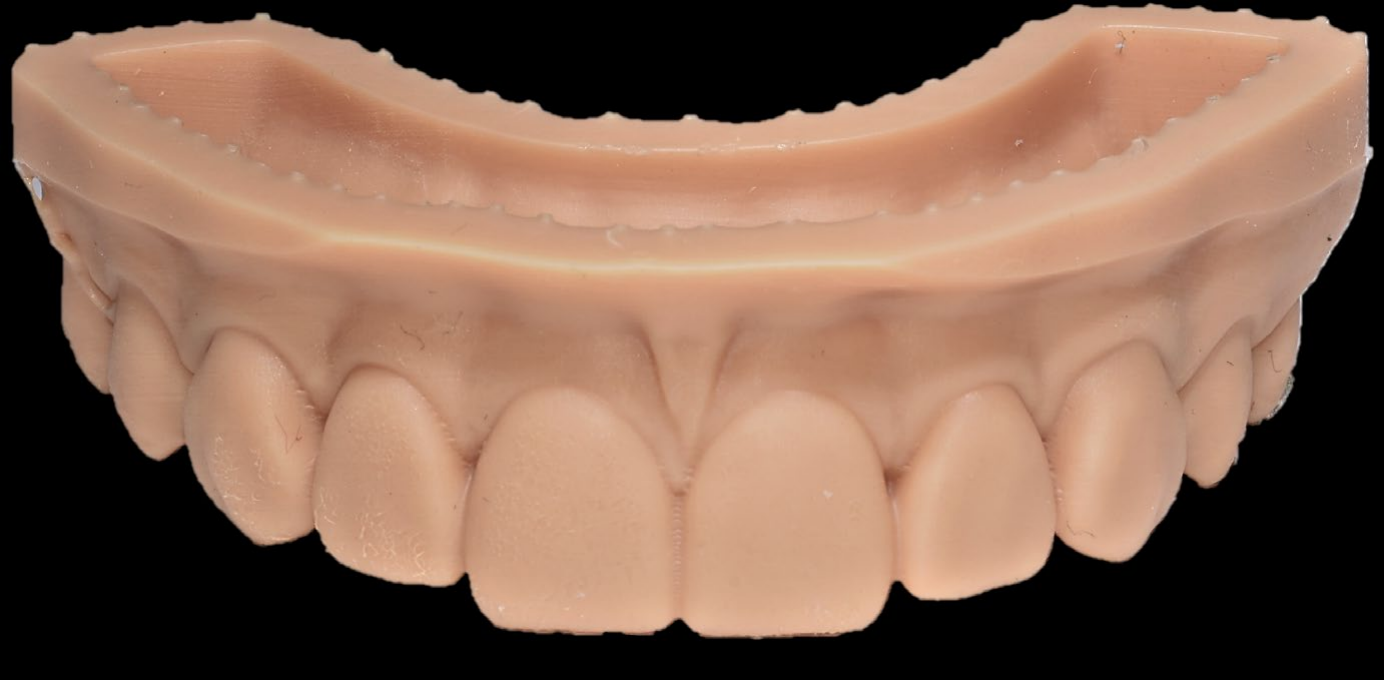
The virtual wax‐up's model from 3D printer

**FIGURE 8:**
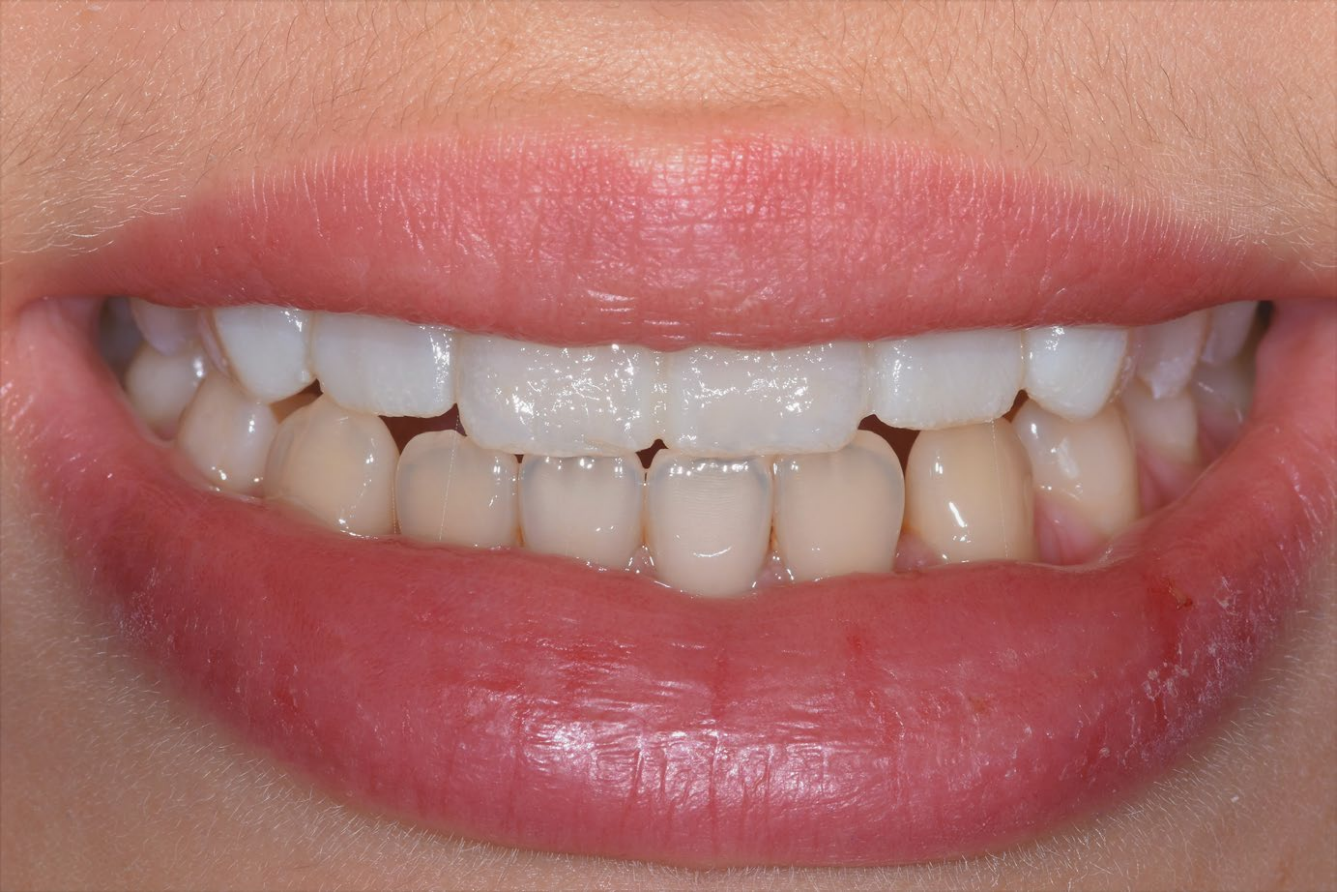
Clinical test of the mock‐up for aesthetics and functionality: detail of the smile

**FIGURE 9:**
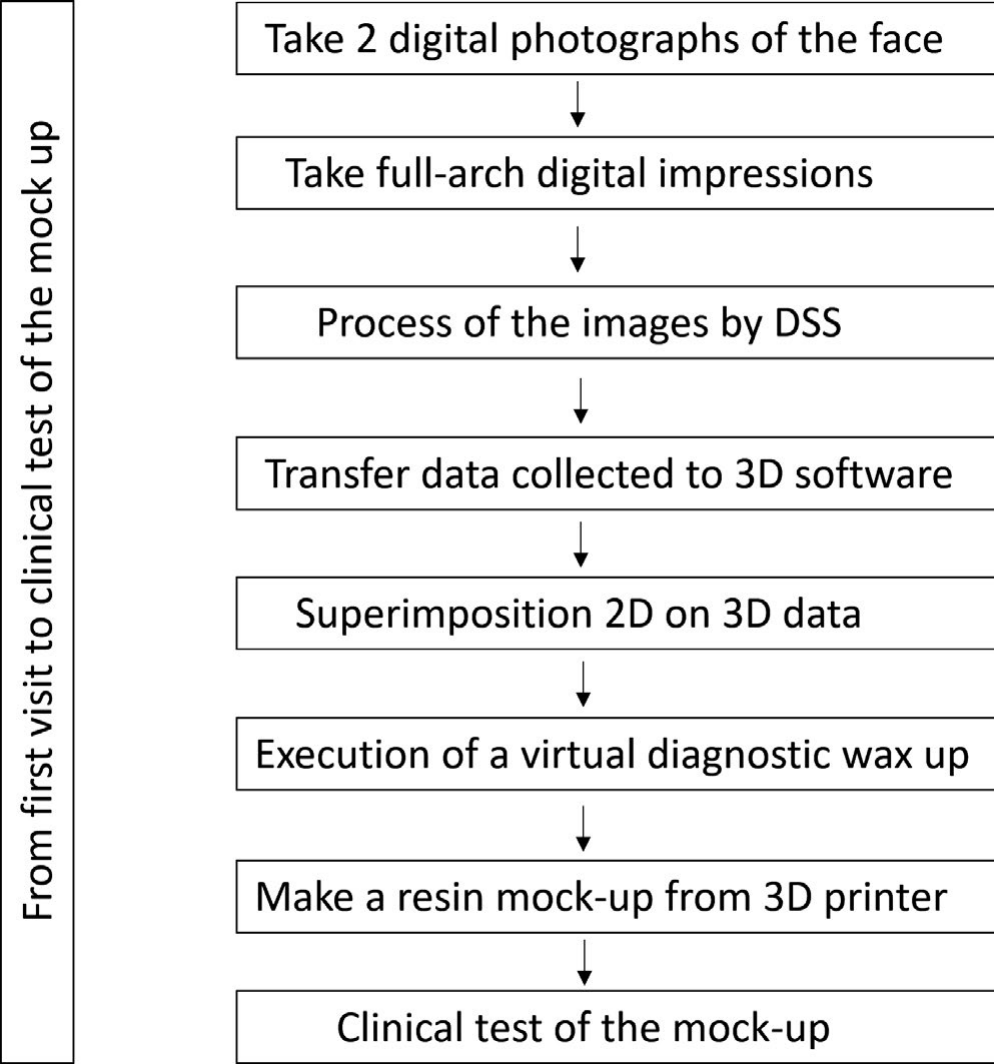
Flow diagram showing main steps from the visit to clinical test of the mock‐up

**FIGURE 10:**
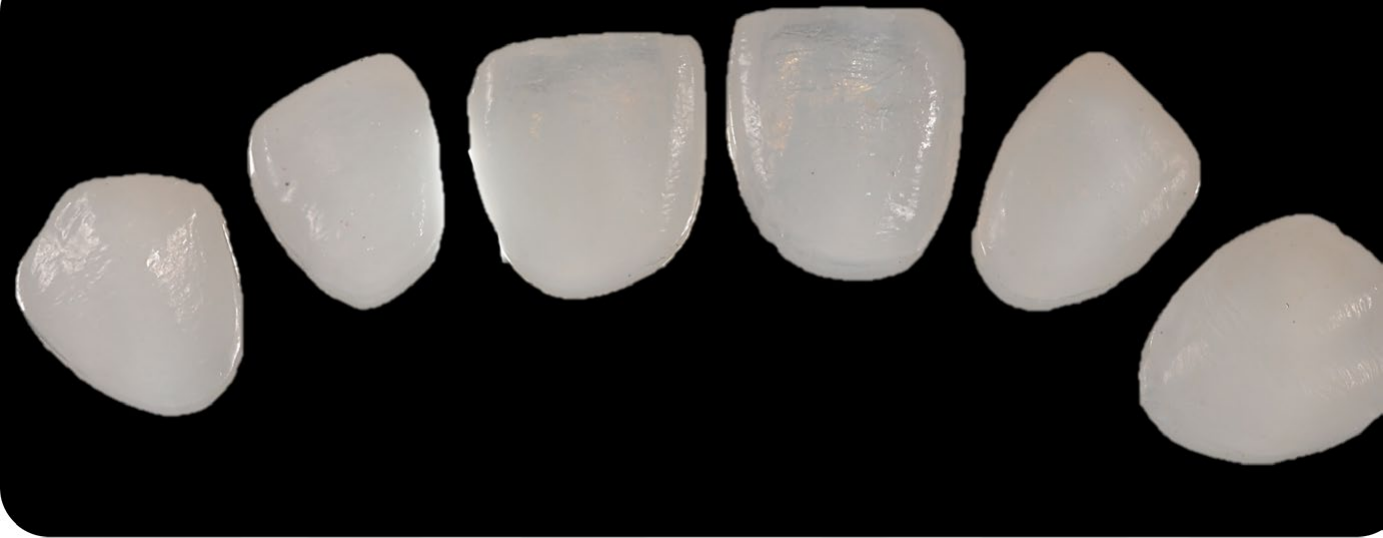
Completed CAD/CAM‐fabricated composite veneers

**FIGURE 11:**
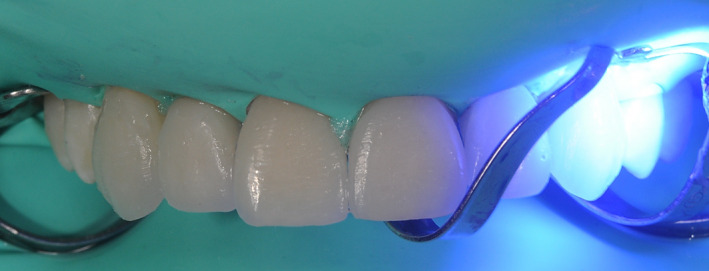
Bonded veneer with rubber dam in place

**FIGURE 12:**
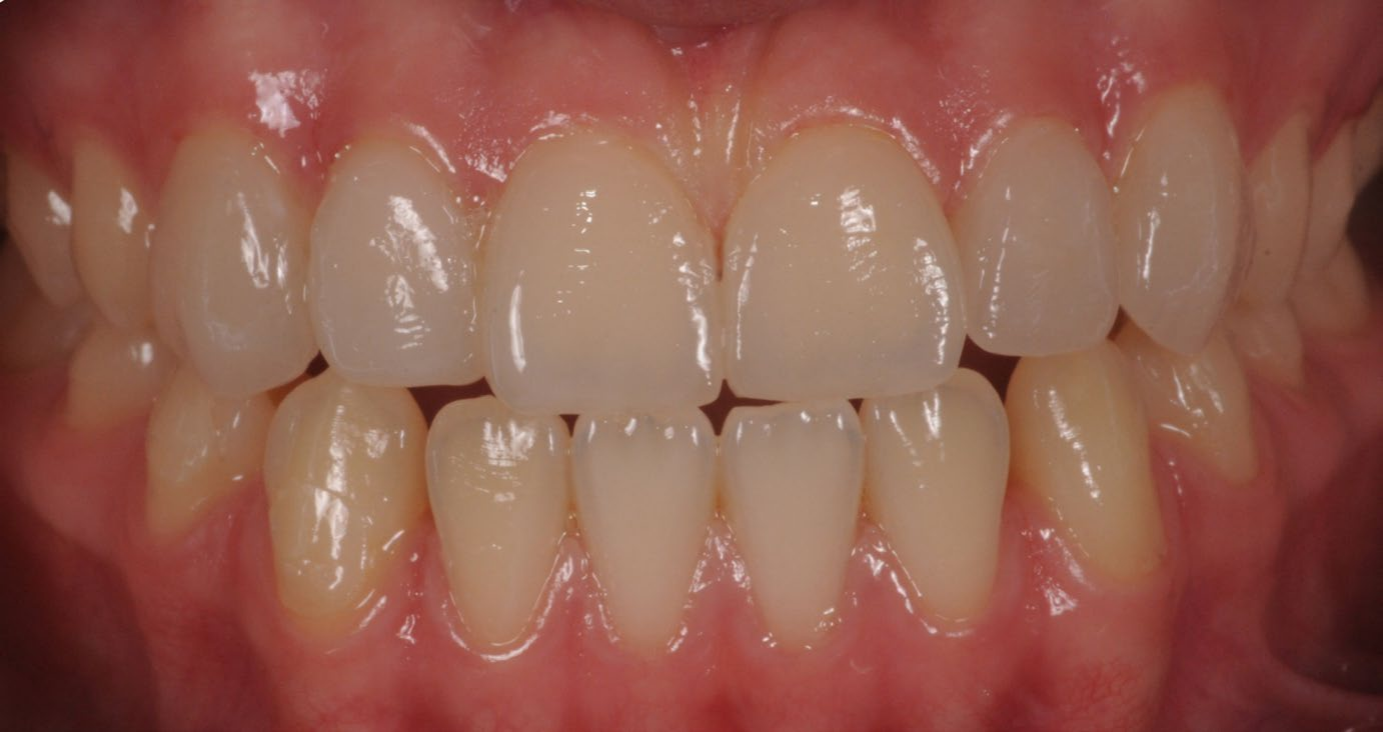
Postoperative intraoral view showing the good aesthetic integration

**FIGURE 13:**
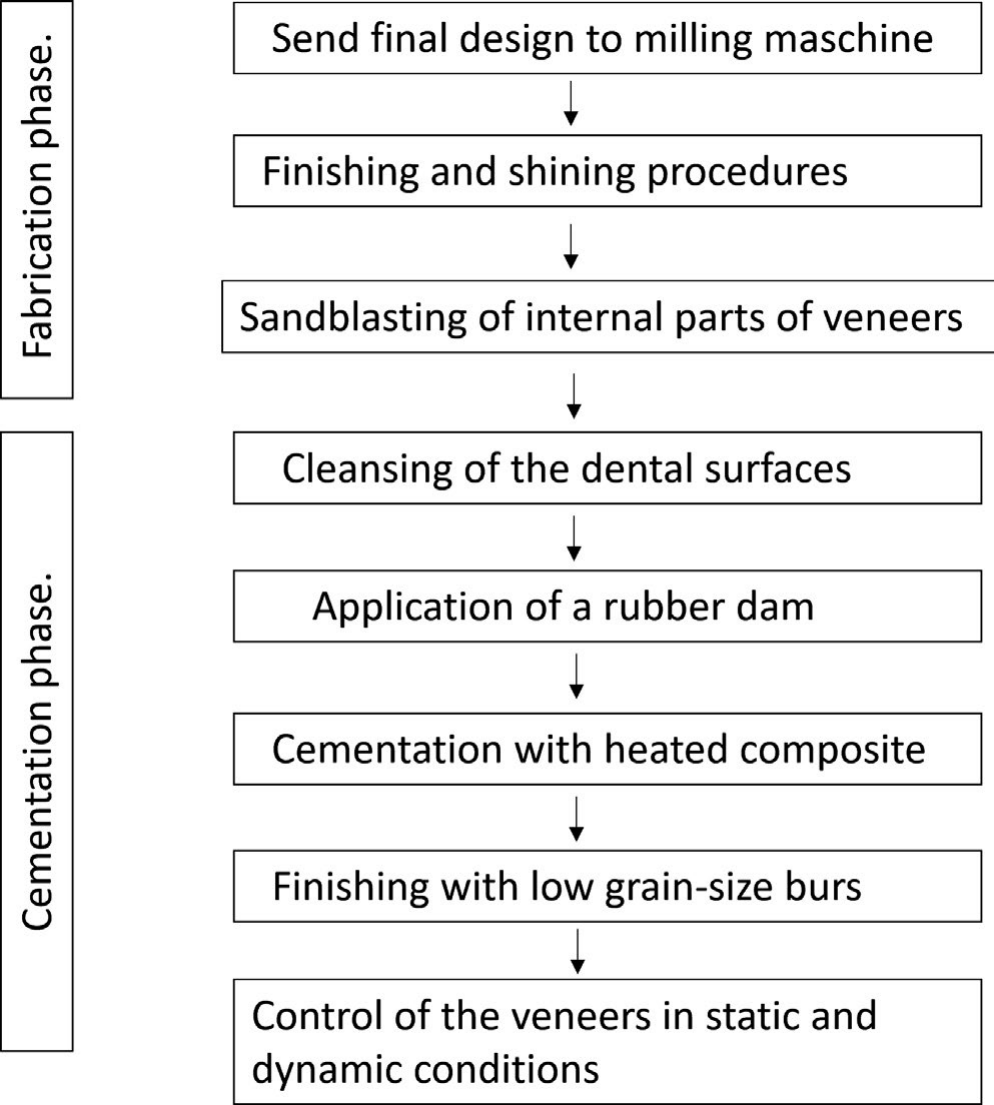
Flow diagram showing main steps for the production and cementation of veneers

**FIGURE 14:**
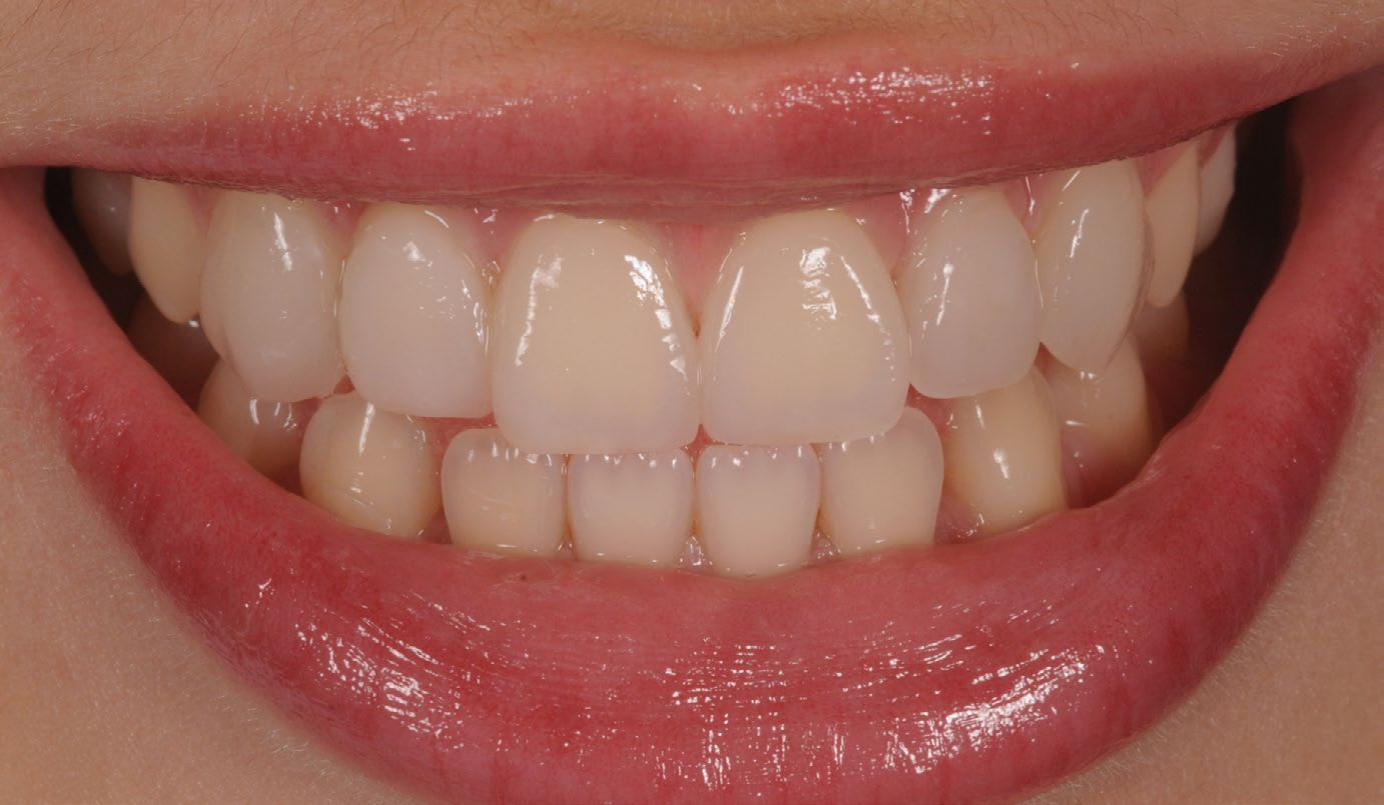
Patient's final smile with proper incisal edge position

**FIGURE 15:**
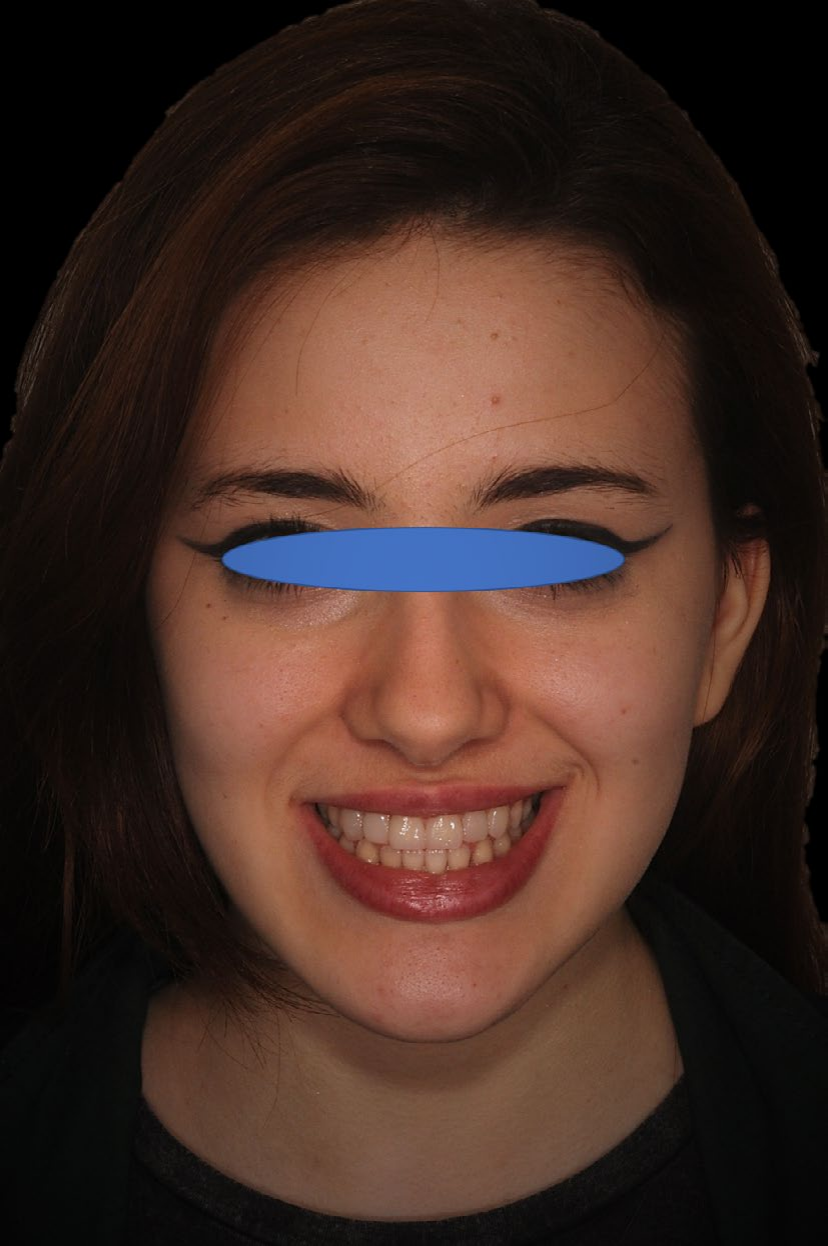
Patient's face at the end of the therapy: veneers permit greatly improved aesthetics

- Taking optical impressions of the dental arches via an intraoral scanner (Trios, 3 Shape A/S, Denmark).
- Processing of the images of the face by a software for a digital preview (DSS, Digital Smile System, Bologna, Italy) to propose virtual planning of the future smile to the patient. Virtual teeth from the software library are chosen according to aesthetic and functional parameters. The preview is shown to the patient for approval or for any changes (Figure 4).
- After approval, transfer of the data collected, including the 2D image with the final aesthetic outlines and the optical impression files, inside a 3D software (Exocad GmbH, Darmstadt, Germany).
- Pairing of the 2D image of the patient's smile with the 3D impression by the 3D software^16^ (Figure 5).
- Execution of a virtual diagnostic wax‐up following the 2D software indications (the outlines represent the contour of the future tooth) and approval by the patient, making use of the 3D libraries or creating customized teeth with tools from Freeform (Exocad® software) (Figure 6).
- Transfer of the wax‐up file to a 3D printer (Formlabs, Formlabs Inc, USA) to make a methacrylic photoreactive resin mock‐up^17^ (Formlabs, Photopolymer Resin, White (GPWH02), Formlabs Inc, USA) to be tested in the patient's oral cavity (as long as thickness is sufficient) or a model (Dental Model Resin; Formlabs) on which a mask can be made to print the mock‐up, when insufficient thicknesses preclude printing of the part, as in this case (Figure 7).
- Clinical testing of the mock‐up for aesthetics and functionality to obtain the patient's final consent (Figure 8). If small changes to the mock‐up are necessary, a new optical impression is done so that the changes made will be in 3D (Figure 9).
- sending of the approved file to a milling device (CORiTEC 250i, Eiterfeld, Germany) onto which a composite disk suited to the clinical situation was positioned: a multilayer disk for high‐thickness veneers or only enamel disks if the thickness is limited (Brilliant Crios HT, Coltene, Switzerland).
- Finishing and shining of the veneers and sandblasting of the internal parts with dedicated sand (Rocatec Plus, 3M ESPE), to increase the bond with the tooth in the post‐cementation phase.^18^
- Sending of the dental veneers for cementation procedures (Figure 10).
- Cleansing of the dental surfaces to be cemented with rubber cups and dedicated cleansing paste
- Sandblasting of the dental surfaces with aluminum oxide to increase veneer retention to the tooth.^19^, ^20^
- Application of a rubber dam and treatment of surfaces for adhesion to the enamel: application of orthophosphoric acid for 30 seconds, and application of the adhesive and polymerization.
- Application of the heated composite material onto the internal part of the veneer and onto the tooth, positioning of the part, removal of any excess, and polymerization (Figure 11).
- Finishing with low grain‐size mills and rubbers, removal of the dam.
- Functionality control of the veneers in static and dynamic conditions (Figures 12, 13, 14 and 15).

## 2 DISCUSSION

In modern digital dentistry, the fundamental steps of the therapeutic flow can be summarized into 3 fundamental phases: acquisition of the images and patient data, processing of the information and production of parts, and clinical application onto patients.^21^, ^22^ Digital instruments and the CAD‐CAM enable development of more predictable and reliable prosthetic workflows. Furthermore, the immediate control of the operational procedures guarantees the transmission of accurate information to the dental laboratory and to all the team involved in the therapy.^23^

The aesthetic demands and expectations of the patients also increase: The digital aesthetic preview thus becomes an expression of everything that the clinician in theory is able to do, legitimizing the patient's demands.^24^

In the described clinical case, the accuracy of the Digital Smile System software was assessed by making customized composite veneers from a totally digital workflow and checking the consistency with the initial project, as described experimentally by other authors.^25^

As previously underscored, acquisition of the images is one of the most delicate phases; use of a reflex camera and a standardized photography method that relies on a calibration instrument, such as the glasses, achieves the patient images that are not only a means for dialogue but also the start of a real and true therapeutic protocol.

Another important workflow phase is the pairing of data inside the 3D software, in particular in the overlapping of the patient image and the optical impression. For successful results, the skills and experience of the operator play a vital role. Often this phase is delegated to the dental technician even though it is not an exclusive skill thereof.

The clinical adequacy of the composite veneers, tested in the patient's oral cavity, is demonstrated by the excellent prosthetic fit. In some situations, management of the production of milled parts is complex due to their low thickness, typical of additional veneers. Furthermore, the presence of any undercuts does not enable the miller to correctly reproduce the superficial details that would be compensated by the composite material in the cementation phase. By contrast, one of the advantages of composite veneers is that they can easily be repaired.

## 3 CONCLUSIONS

Use of a software that can digitally plan aesthetic and functional rehabilitation of the smile notably improves the clinical workflow. The advantages of digital planning of a prosthetic case are mainly the efficacy of communication with the patient, who can be shown a preview of the result, and with the dental laboratory, providing all information necessary to make the parts. The clinician, through 2D and 3D software, associated with digital image editing, can process the images, assessing every specific clinical‐aesthetic demand and showing the patient a preview of the result. Said data, in the case of totally digital workflow, can be transmitted directly to the dental laboratory for fabrication of the parts, reducing the time and costs typical of traditional planning. Furthermore, the progress obtained by modern digital dentistry tends to increasingly reduce operator errors.

In this study, the reliability of the virtual project for making the customized veneers was assessed through a totally digital workflow. Although the result is satisfactory and is faithful with the digital preview, it should be underscored that the operator's skill at performing the digital passages is still an aspect not to be undervalued. Even today, technology cannot completely replace the work of an expert, in our view.

The result achieved in the performance of this case should be confirmed on a broader sample of subjects.

## CONFLICTS OF INTEREST

The author reports no conflicts of interest.

## AUTHOR CONTRIBUTIONS

LO, TV, MLS, and LL: conceptualized the study. LO and MO: designed the methodology. LO and TV: provided the software. TV: provided the resources and supervised the study. LO: involved in data curation and wrote the original draft of the manuscript. LO and TV: wrote, reviewed, and edited the manuscript. LO, TV, and MLS: visualized the study. MO: made the laminate veneers.

## CONSENT STATEMENT

The patient has provided consent for the use of photographs and clinical records for publication.

## References

[1] Abad‐Coronel C , Naranjo B , Valdiviezo P . Adhesive systems used in indirect restorations cementation: review of the literature. Dent J. 2019;7(3):71. 10.3390/dj7030071 PMC6784471 31266163

[2] Korkut B , Yanıkoğlu F , Günday M . Direct composite laminate veneers: three case reports. J Dent Res Dent Clin Dent Prospects. 2013;7(2):105. 23875090 10.5681/joddd.2013.019 PMC3713859

[3] Celik N , Yapar MI , Taşpınar N , Seven N . The effect of polymerization and preparation techniques on the microleakage of composite laminate veneers. Contemp Clin Dent. 2017;8(3):400. 29042725 10.4103/ccd.ccd_46_17 PMC5643997

[4] Dukic W , Dukic OL , Milardovic S , Delija B . Clinical evaluation of indirect composite restorations at baseline and 36 months after placement. Oper Dent. 2010;35(2):156‐164. 20420058 10.2341/09-133-C

[5] Pini NP , Aguiar FHB , Lima DANL , Lovadino JR , Terada RSS , Pascotto RC . Advances in dental veneers: materials, applications, and techniques. Clin Cosmet Investig Dent. 2012;4:9. 10.2147/CCIDEN.S7837 PMC3652364 23674920

[6] Jain V , Das TK , Pruthi G , Shah N , Rajendiran S . Comparative evaluation of effects of bleaching on color stability and marginal adaptation of discolored direct and indirect composite laminate veneers under in vivo conditions. J Indian Prosthodont Soc. 2015;15(1):46. 26929486 10.4103/0972-4052.155038 PMC4762281

[7] Stawarczyk B , Liebermann A , Eichberger M , Güth JF . Evaluation of mechanical and optical behavior of current esthetic dental restorative CAD/CAM composites. J Mech Behav Biomed Mater. 2016;55:1‐11. 10.1016/j.jmbbm.2015.10.004 26519658

[8] Van Meerbeek B , Peumans M , Poitevin A , et al. Relationship between bond‐strength tests and clinical outcomes. Dent Mater. 2010;26(2):e100‐e121. 20006379 10.1016/j.dental.2009.11.148

[9] Veneziani M . Ceramic laminate veneers: clinical procedures with a multidisciplinary approach. Int J Esthet Dent. 2017;12(4):426‐448. 28983530

[10] Cervino G , Fiorillo L , Arzukanyan AV , Spagnuolo G , Cicciù M . Dental restorative digital workflow: digital smile design from aesthetic to function. Dent J (Basel). 2019;7(2):30. Review. 10.3390/dj7020030 PMC6632039 30925698

[11] Jokstad A . Computer‐assisted technologies used in oral rehabilitation and the clinical documentation of alleged advantages—a systematic review. J Oral Rehabil. 2017;44:261‐290. 28109024 10.1111/joor.12483

[12] Sanchez‐Lara A , Chochlidakis K , Lampraki E , Molinelli R , Molinelli F , Ercoli C . Comprehensive digital approach with the digital smile system: a clinical report. J Prosthet Dent. 2019;121(6):871‐875. 30885588 10.1016/j.prosdent.2018.10.012

[13] Ortensi L , Vitali T , Bonfiglioli R , Grande F . New tricks in the preparation design for prosthetic ceramic laminate veeners. Prosthesis. 2019;1(1):29‐40.

[14] Stefani R , Caviggioli I , Molinelli F , Ortensi L . L’impiego delle tecnologie digitali nella diagnosi protesica e nella realizzazione della protesi. Il Dentista Moderno. Ottobre; 2012.

[15] Lavorgna L , Vitali T , Caviggioli I , Ortensi L . Fully digital workflow for an implant retained overdenture by digital smile project to guided surgery and prosthetic rehabilitation. Int J Sci Res. 2018;7:12.

[16] Stanley M , Paz AG , Miguel I , Coachman C . Fully digital workflow, integrating dental scan, smile design and CAD‐CAM: case report. BMC Oral Health. 2018;18(1):134. 30086753 10.1186/s12903-018-0597-0 PMC6081948

[17] Cattoni F , Mastrangelo F , Gherlone EF , Gastaldi G . A new total digital smile planning technique (3D‐DSP) to fabricate CAD‐ CAM mockups for esthetic crowns and veneers. Int. J Dent. 2016;2016:1‐5. 10.1155/2016/6282587 PMC4958427 27478442

[18] Iwasaki T , Komine F , Fushiki R , Kubochi K , Shinohara M , Matsumura H . Shear bond strengths of an indirect composite layering material to a tribochemically silica‐coated zirconia framework material. Dent Mater J. 2016;35(3):461‐469. 27252003 10.4012/dmj.2015-311

[19] Cal‐Neto JP , Castro S , Moura PM , Ribeiro D , Miguel JAM . Influence of enamel sandblasting prior to etching on shear bond strength of indirectly bonded lingual appliances. Angle Orthod. 2011;81(1):149‐152. 20936968 10.2319/050210-237.1 PMC8926354

[20] Elsaka SE . Bond strength of novel CAD/CAM restorative materials to self‐adhesive resin cement: the effect of surface treatments. J Adhes Dent. 2014;16(6). 531–540. 25516881 10.3290/j.jad.a33198

[21] Mangano F , Shibli JA , Fortin T . Digital dentistry: new materials and techniques. Int J Dent. 2016;2016:1‐2. 10.1155/2016/5261247 PMC5093293 27840638

[22] Fasbinder D .Digital dentistry: innovation for restorative treatment. Compendium of continuing education in dentistry (Jamesburg, N.J.: 1995). 31 Spec No 4. 2–11; quiz 12. 21049823

[23] Coachman C , Calamita MA , Sesma N . Dynamic documentation of the smile and the 2D/3D digital smile design process. Int J Periodontics Restorative Dent. 2017;37(2):183‐193. 28196157 10.11607/prd.2911

[24] Omar D , Duarte C . The application of parameters for comprehensive smile esthetics by digital smile design programs: A review of literature. Saudi Dent J. 2018;30(1):7‐12. 30166865 10.1016/j.sdentj.2017.09.001 PMC6112329

[25] Lavorgna L , Cervino G , Fiorillo L , et al. Reliability of a virtual prosthodontic project realized through a 2D and 3D photographic acquisition: an experimental study on the accuracy of different digital systems. Int J Environ Res Public Health. 2019;16(24):5139. 10.3390/ijerph16245139 PMC6950125 31888225

